# Further investigation of the characteristics and biological function of *Eimeria tenella* apical membrane antigen 1

**DOI:** 10.1051/parasite/2020068

**Published:** 2020-12-11

**Authors:** Qingjie Wang, Qiping Zhao, Shunhai Zhu, Bing Huang, Shuilan Yu, Shanshan Liang, Haixia Wang, Huanzhi Zhao, Hongyu Han, Hui Dong

**Affiliations:** Key Laboratory of Animal Parasitology of the Ministry of Agriculture, Shanghai Veterinary Research Institute, CAAS 200241 Shanghai PR China

**Keywords:** Apicomplexa, *Eimeria tenella*, Apical membrane antigen, iTRAQ, DF-1 cells

## Abstract

Apical membrane antigen 1 (AMA1) is a type I integral membrane protein that is highly conserved in apicomplexan parasites. Previous studies have shown that *Eimeria tenella* AMA1 (EtAMA1) is critical for sporozoite invasion of host cells. Here, we show that EtAMA1 is a microneme protein secreted by sporozoites, confirming previous results. Individual and combined treatment with antibodies of EtAMA1 and its interacting proteins, *E. tenella* rhoptry neck protein 2 (EtRON2) and *Eimeria*-specific protein (EtESP), elicited significant anti-invasion effects on the parasite in a concentration-dependent manner. The overexpression of EtAMA1 in DF-1 cells showed a significant increase of sporozoite invasion. Isobaric tags for relative and absolute quantitation (iTRAQ) coupled with LC-MS/MS were used to screen differentially expressed proteins (DEPs) in DF-1 cells transiently transfected with EtAMA1. In total, 3953 distinct nonredundant proteins were identiﬁed and 163 of these were found to be differentially expressed, including 91 upregulated proteins and 72 downregulated proteins. The DEPs were mainly localized within the cytoplasm and were involved in protein binding and poly(A)-RNA binding. KEEG analyses suggested that the key pathways that the DEPs belonged to included melanogenesis, spliceosomes, tight junctions, and the FoxO and MAPK signaling pathways. The data in this study not only provide a comprehensive dataset for the overall protein changes caused by EtAMA1 expression, but also shed light on EtAMA1’s potential molecular mechanisms during *Eimeria* infections.

## Abstract (fr)

L’antigène 1 de la membrane apicale (AMA1) est une protéine membranaire intégrale de type I hautement conservée chez les parasites Apicomplexa. Des études antérieures ont montré que l’AMA1 d’*Eimeria tenella* (EtAMA1) était importante pour l’invasion des cellules hôtes par les sporozoïtes. Nous montrons ici qu’EtAMA1 est une protéine des micronèmes sécrétée par les sporozoïtes, confirmant les résultats précédents. Un traitement individuel et combiné avec des anticorps d’EtAMA1 et de ses protéines en interaction, la protéine 2 du cou des rhoptries d’*E. tenella* (EtRON2) et la protéine spécifique d’*Eimeria* (EtESP), a provoqué des effets anti-invasion significatifs et dépendants de la concentration sur le parasite. La surexpression d’EtAMA1 dans les cellules DF-1 a montré une augmentation significative de l’invasion par les sporozoïtes. Des marqueurs isobares pour la quantification relative et absolue (iTRAQ) couplés à LC-MS/MS ont été utilisés pour cribler des protéines exprimées différentiellement (PED) dans des cellules DF-1 transfectées de manière transitoire avec EtAMA1. Au total, 3953 protéines non redondantes distinctes ont été identiﬁées et 163 d’entre elles se sont révélées exprimées de manière différentielle, dont 91 régulées à la hausse et 72 régulées à la baisse. Les PED étaient principalement localisées dans le cytoplasme et étaient impliquées dans la liaison aux protéines et la liaison au poly (A)-ARN. Les analyses de KEEG ont suggéré que les voies clés auxquelles appartenaient les PED comprenaient la mélanogenèse, les épissosomes, les jonctions étroites et les voies de signalisation FoxO et MAPK. Les données de cette étude fournissent non seulement un ensemble de données complet pour les modifications globales des protéines causées par l’expression d’EtAMA1, mais mettent également en lumière les mécanismes moléculaires potentiels d’EtAMA1 pendant les infections par *Eimeria*.

## Introduction

Parasites of the phylum Apicomplexa are responsible for some of the most devastating and burdensome diseases to humans and animals worldwide, including malaria, cryptosporidiosis, toxoplasmosis, and coccidiosis [5]. Host-cell invasion is an essential step in the life cycle and pathogenesis of apicomplexan parasites. A conserved feature of host cell invasion by these protozoan pathogens is the formation of an intimate and circumferential contact area with the host cell, called the moving junction (MJ) [1]. Initially formed at the apical tip of the invading parasite, the MJ is important for successful invasion as it represents a ring-like region of contact between the surfaces of the invading parasite and the host cell as the invaginated host plasma membrane is forced inward by the penetrating parasite [38].

Previous reports on *Toxoplasma* and *Plasmodium* detailed the composition of the MJ complex, which mainly includes rhoptry neck proteins (RONs) and apical membrane antigen1 (AMA1) [2, 3, 7]. More precisely, the parasite exports the microneme protein, AMA1, to its own surface and the rhoptry neck RON2 protein is the receptor inserted into the host cell with other RON partners [28]. Of all the MJ components, AMA1 is the best characterized. Initially identiﬁed in *P. knowlesi* nearly 40 years ago [13], AMA1 is a type I integral membrane protein that is highly conserved in apicomplexan parasites [6]. Numerous lines of evidence have found that AMA1 mediates invasion or attachment of *Toxoplasma* tachyzoites [24], *Plasmodium* merozoites [25], *Neospora* tachyzoites [45], and *Babesia* merozoites [33] to their respective host cells.

*Eimeria tenella* is widely considered to be the most economically relevant and well-known of the seven *Eimeria* species that cause coccidiosis in chickens [10]. *Eimeria tenella* has a complex life cycle that includes two major asexual developmental stages, including sporozoites and the merozoites [21]. The expressed sequence tags (ESTs) of the sporozoites and the merozoites were analyzed, and some ESTs exhibited homology with AMA1 [27]. Proteomic comparisons of four *E. tenella* life-cycle stages found that EtAMA1 was detected only in sporozoites [20]. Jiang et al. characterized EtAMA1 and found that it was expressed at higher levels in sporozoites than in other developmental stages [18]. Speciﬁc EtAMA1 antibodies, recombinant proteins, or binding peptides can significantly inhibit sporozoite invasion of host cells [18, 23, 29].

EtAMA1 can interact with *E. tenella* rhoptry neck protein 2 (EtRON2), microneme protein 2 (EtMIC2), and an *Eimeria*-specific protein (EtESP) (GenBank accession No. XM_013373193) [41, 44]. Other putative EtAMA1-interacting proteins include *E. tenella* putative cystathionine beta-synthase, four conserved hypothetical proteins (one in the serine/threonine protein kinase family), and seven unknown proteins, but other putative proteins must be further identified [16]. Thus, this study further characterized the function and mechanism of EtAMA1 during *Eimeria* host cell invasion.

## Materials and methods

### Ethics statement

All animal procedures were approved by the Animal Ethics Committee of the Shanghai Veterinary Institute, Chinese Academy of Agricultural Science. Experiments were conducted in accordance with animal ethics guidelines and approved protocols.

### Parasites, birds, and cells

The Shanghai strain of *E. tenella* was maintained and propagated by regular *in vivo* passage through 2-week-old Yellow chickens reared under specific pathogen-free conditions [22]. Unsporulated oocysts, sporulated oocysts, sporozoites, and second-generation merozoites were collected and purified, as described previously [34]. The chicken embryo fibroblast cell line, DF-1, was cultured in complete medium (CM) (Dulbecco’s Modified Eagle’s Medium (DMEM) (Gibco BRL, Paisley, UK) containing 10% fetal calf serum (FCS) (Gibco), and 100 U/mL penicillin/streptomycin (Gibco).

### EtAMA1 secretion assays

To test the secretion of *Et*AMA1, *E. tenella* microneme 2 protein (EtMIC2) was used as the experimental control [35, 43]. A total of 4 × 10^6^ fresh sporozoites were resuspended in 100 μL PBS or complete medium and incubated for 2 h at 4 °C or at 41 °C. Then 5 μM, 10 μM, or 20 μM of staurosporine (Sigma-Aldrich, St. Louis, MO, USA), a protein kinase inhibitor known to speciﬁcally inhibit microneme secretion [9, 19], dissolved in dimethyl sulfoxide (DMSO) or solvent control (DMSO), was added, as described previously [19]. Sporozoites were then pelleted by centrifugation for 5 min at 6000 ×*g* (Eppendorf Centrifuge 5810R). Supernatants containing excretory-secretory antigens (ESAs) were harvested and EtAMA1 and EtMIC2 secretion was analyzed by western blotting with anti-rEtAMA1 or anti-rEtMIC2 antibodies, respectively [18, 43].

### Immunoﬂuorescence analysis

To identify co-localization of EtAMA1 and its interaction proteins, EtRON2 and EtESP, in *E. tenella*, the purified sporozoites were transferred to glass slides and air-dried. Sporozoites were fixed in 2% paraformaldehyde in PBS, air-dried, and permeabilized with 1% Triton X-100 in PBS for 15 min. The slides were blocked with 2% (w/v) bovine serum albumin in PBS for 2 h at 37 °C and incubated with polyclonal anti-rEtAMA1 mouse serum, and anti-rEtESP or anti-rEtRON2 rabbit serum [43] diluted in PBS (1:100) at 37 °C for 2 h. Slides were then incubated with Alexa Fluor™ 488 chicken anti-rabbit IgG or Alexa Fluor™ 647 chicken anti-mouse IgG secondary antibodies (1:500 dilution) for 1 h at 37 °C. Nuclei were stained with 2-(4-amidinophenyl)-6-indolecarbamidine dihydrochloride (Beyotime) (10 mg/mL) for 20 min at room temperature. After each step, the slides were washed three times for 10 min each with PBS containing 0.05% Tween 20. Slides were then mounted using Fluoromount Aqueous Mounting Medium (Sigma-Aldrich) and observed under a laser scanning confocal microscope (Zeiss, Germany).

### Inhibition assays

A neutralization assay with antibodies was used to assess the potential involvement of EtAMA1 and its interaction proteins, EtRON2 and EtESP, in host cell invasion by *E. tenella* sporozoites. The ani-rEtAMA1, ani-rEtRON2, and ani-rEtESP antibodies were purified using protein A + G agarose (Beyotime), according to the manufacturer’s instructions. DF-1 cells (2 × 10^5^ cells per well) were cultivated in 24-well plates (Corning, NY, USA) in CM for 12 h at 37 °C and 5% CO_2_. Freshly purified sporozoites were marked using carboxyfluorescein diacetate succinimidyl ester (CFSE; Beyotime Biotechnology), according to the manufacturer’s instructions. The labelled sporozoites were preincubated at 37 °C for 2 h with the purified anti-rEtAMA1, rEtESP or rEtRON2 IgG, or combinations of anti-rEtAMA1 and rEtESP IgGs (with a ratio of 1:1), or anti-rEtAMA1 and rEtRON2 IgGs (with a ratio of 1:1) at concentrations of 100, 200, 400, or 600 μg/mL [26]. The same amount of IgG from naive rabbit serum (Sigma-Aldrich) and IgG-untreated sporozoites were used as controls. Sporozoites were added to DF-1 cells for 8 h at 41 °C and 5% CO_2_ with a sporozoite/cell ratio of 3:1. The cells were washed, trypsinized, harvested, and analyzed using a flow cytometer (model Cytomics FC500; Beckman Coulter, USA). The infected cells, non-infected cells and free sporozoites were gated using the software RXP for subsequent delineation and counting of the infected (containing the labeled sporozoites) and non-infected (fluorescence-free) cells. Three replicates were performed for all the tests, and a total of three tests were conducted. The percentage of infected cells in the presence or absence of inhibitory antibodies was calculated as previously described [17].

### *In vitro* sporozoite invasion of transiently EtAMA1-transfected DF-1 cells

The extracellular domain I of EtAMA1 (GenBank accession No. LN610018.1) was amplified using the primers: 5′–GCGAATTCATGGGGCCACCCCCGTCTTTGGCAAAAAC–3′ and 5′–GCAGA TCTGCACTTGG TCTCCCAGTCG–3′ containing EcoRI sand BgIII restriction sites (underlined). The PCR products were ligated into the pcDNA3.1(+) vector and designated as pcDNA3.1(+)-*Et*AMA1. The constructed plasmid was identified by sequencing and purified using a Qiagen Plasmid Giga Kit (Qiagen). The plasmid concentration was determined by spectrophotometry at 260 nm.

A monolayer of 80–90% confluent DF-1 cells in six-well plates was transfected with pcDNA3.1(+)-*Et*AMA1 or pcDNA3.1(+) using Lipofectamine^®^ (Invitrogen). Six hours later, the DNA-transfection reagent mixture was replaced by opti-MEM. At 48 h post-transfection, the expression of *Et*AMA1 in transfected DF-1 cells was confirmed by indirect fluorescent antibody testing (IFA) (Dong et al. [15]). The proliferation of transfected cells was determined using Cell Counting Kit-8 (Beyotime), according to the manufacturer’s protocol.

The pcDNA3.1(+)-*Et*AMA1 or pcDNA3.1(+) transfected DF-1 cells were invaded by fluorescently-labeled *E. tenella* sporozoites with CFSE at a ratio of three sporozoites per cell for 8 h at 41 °C, and then subjected to flow cytometry to determine the invasion rate. The sporozoite-infected un-treated DF-1 cells were the control group.

### iTRAQ-based comparative proteomic analysis of transiently EtAMA1-transfected DF-1 cells

The pcDNA3.1(+)-*Et*AMA1-transfected and empty vector pcDNA3.1(+)-transfected DF-1 cells were seeded into T25 culture ﬂasks and cultured in a 5% CO_2_ incubator at 37 °C for 48 h. Each group was treated with three independent biological replicates. The transfected cells were washed three times with chilled PBS, collected with cell scrapers, and homogenized in lysis buffer (4% SDS, 1 mM DTT, 150 mM Tris-HCl pH 8.0, protease inhibitor), then incubated for 3 min in boiling water and sonicated twice on ice, and clariﬁed by centrifugation at 16,000 ×*g* at 25 °C for 10 min. Protein content was determined using the BCA protein assay reagent (Beyotime). Protein digestion, iTRAQ labeling, peptide fractionation using strong cation exchange (SCX) chromatography, and liquid chromatography (LC)-electrospray ionization tandem MS (MS/MS) analyses with Q Exactive were conducted as previously described [4, 40, 42].

MS/MS spectra were searched using the Mascot 2.2 engine against the UniProt *Gallus gallus* database (36,539 sequences, downloaded on June 9, 2018) and the decoy database. For identiﬁcation, the following options were used. Peptide mass tolerance = 20 ppm, MS/MS tolerance = 0.1 Da, enzyme = trypsin, Missed cleavage = 2, fixed modiﬁcation: carbamidomethyl (C), iTRAQ8plex (K), iTRAQ8plex(N-term), variable modiﬁcation: oxidation (M), and FDR ≤ 0.01. To define up- and downregulated proteins, the levels for significantly differentially expressed proteins (DEPs) were set at a fold change > 1.2 and < 0.83, respectively, with a *p*-value < 0.05 [32, 47].

The Gene Ontology (GO) program, Blast2GO (https://www.blast2go.com/), was used to annotate DEPs to create histograms of GO annotations. For pathway analysis, the DEPs were mapped to the terms in the KEGG (Kyoto Encyclopedia of Genes and Genomes) database using the KAAS program (http://www.genome.jp/kaas-bin/kaas_main).

### Statistical analysis

All data were analyzed using SPSS version 20.0 for Windows (IBM, Armonk, NY, USA) and GraphPad Prism version 6.0 (GraphPad, La Jolla, CA, USA).

The significance of differences in sporozoite invasion rates or inhibitory percentages between the groups was evaluated by one-way analysis of variance (ANOVA) and the mean values were compared using Duncan’s multiple range test. Differences were considered significant and extremely significant if *p* < 0.05 and < 0.01, respectively.

## Results

### EtAMA1 is secreted by micronemes

**Figure 1:**
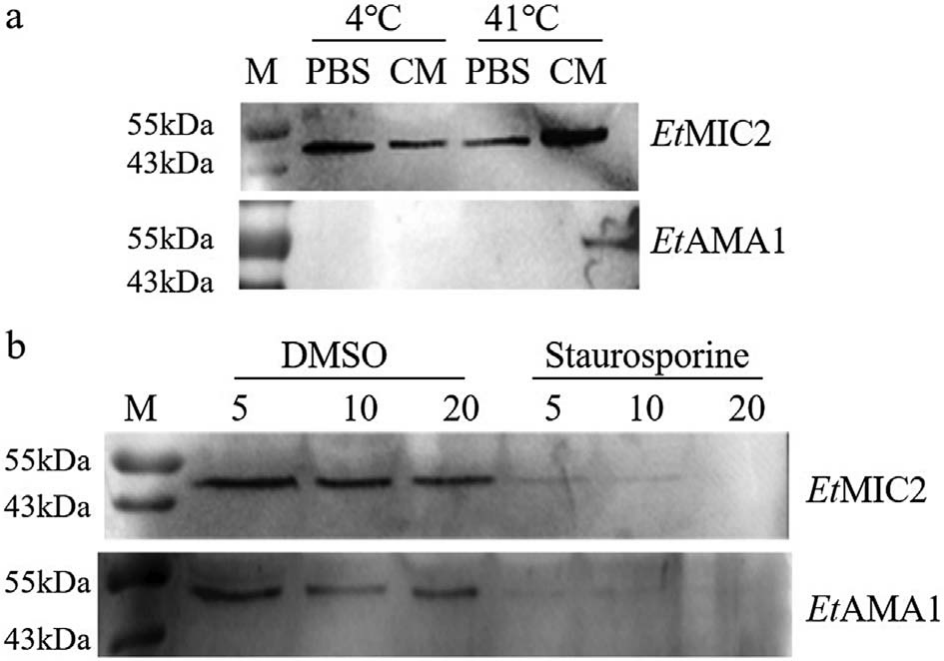
EtAMA1 is secreted by micronemes. (a) EtAMA1 secretion is FCS- and temperature-dependent. Fresh sporozoites were incubated in PBS or complete medium (CM) at 4 °C or 41 °C for 2 h. Supernatants containing excretory-secretory antigens (ESAs) were harvested and analyzed by western blotting to detect EtAMA1 and EtMIC2. (b) EtAMA1 secretion is inhibited by staurosporine. Sporozoites were incubated in CM with various concentrations of staurosporine or DMSO at 41 °C for 2 h. Supernatants containing ESAs was harvested and analyzed by western blotting to detect EtAMA1 and EtMIC2.

To determine whether EtAMA1 is a secreted protein, sporozoites were incubated in PBS and CM at 4 °C or 41 °C, respectively. Western blotting showed that EtAMA1 was secreted when sporozoites were incubated at 41 °C in CM, and EtMIC2 was secreted in PBS and CM at 4 °C or 41 °C (Fig. 1a). Therefore, EtAMA1 secretion was stimulated by FCS and is temperature-dependent.

To demonstrate that EtAMA1 secretion was dependent on the micronemal pathway, staurosporine was used as this protein kinase inhibitor speciﬁcally inhibits micronemal secretion [9, 19]. In sporozoites treated with staurosporine, secretion of EtAMA1 or EtMIC2 in the supernatants decreased in a concentration-dependent manner with increases in inhibitor concentrations (Fig. 1b).

### Co-localization of *Et*AMA1, and EtESP and EtRON2 in parasites

**Figure 2:**
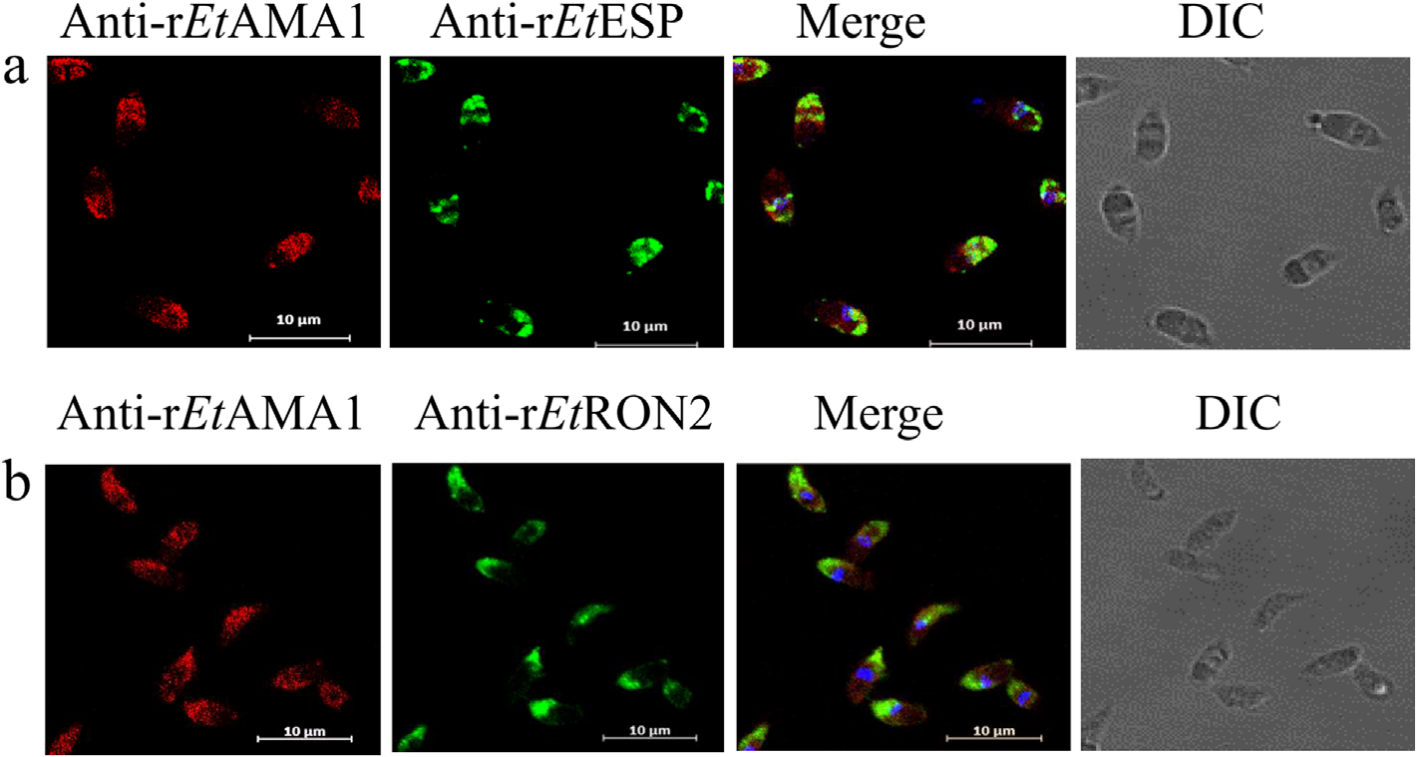
Colocalization of EtAMA1, EtESP, and EtRON2 in sporozoites by indirect immunoﬂuorescence. Parasites were immunostained with anti-rEtAMA1, and anti-rEtESP or anti-rEtRON2 antibodies, visualized with FITC (green) and counter-stained with DAPI (blue). Scale bar, 10 μm.

The locations of EtAMA1 and its interacting proteins, EtESP and EtRON2, were detected in sporozoites incubated in culture medium. IFAs showed that EtAMA1, EtESP and EtRON2 were distributed to the apical end of sporozoites (Figs. 2a and 2b), indicating that the three proteins function in the same location.

### *In vitro* invasion inhibition assay

**Figure 3:**
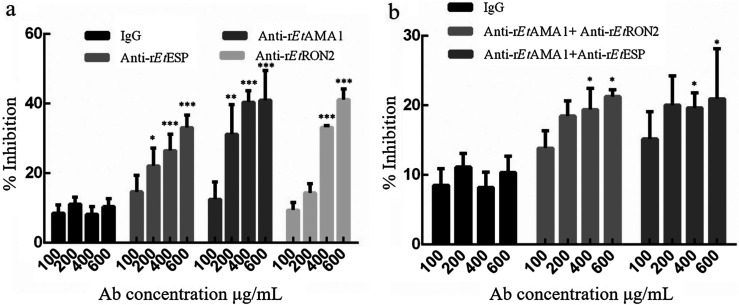
Inhibition of sporozoite invasion *in vitro* by antibodies against rEtAMA1, rEtESP, and rEtRON2. (a) Invasion-inhibition activities of single antibodies. Anti-rEtAMA1, rEtESP, and rEtRON2 rabbit anti-serum against recombinant EtAMA1, EtESP and EtRON2 protein, respectively; IgG, normal rabbit serum. (b) Invasion-inhibition activities of antibody combinations. Combinations of anti-rEtAMA1 and anti-rEtESP or anti-rEtRON2 were added at a ratio of 1:1 to generate a gradient concentration of IgG. All assays were performed in triplicate. *p < 0.05, **p < 0.01 and ***p < 0.001, as determined by the Student’s *t*-test versus the non-immunized IgG groups at the same concentration.

To study the ability of antibodies to block sporozoite invasion in DF-1 cells, anti-rEtAMA1, anti-rEtESP and anti-rEtRON2 IgGs with different concentrations were added to sporozoites and incubated at 37 °C for 2 h. All three antibodies demonstrated high concentration-dependent inhibitory effects on sporozoite invasion into DF-1 cells compared to non-immunized IgGs where minimal changes in invasion were observed. When the antibody concentration was 600 g/mL, the inhibition of invasion rates in the EtAMA1, EtESP, and EtRON2 antibody-treatment groups were 40.9%, 33.1%, and 41.1%, respectively, which were significantly higher than that of non-immunized IgGs (*p* < 0.001) (Fig. 3a). When combinations of anti-rEtAMA1 and anti-rEtESP, or anti-rEtAMA1 and anti-rEtRON2 were used, an inhibitory effect was also observed, but the level of inhibition was lower than that of the individual antibodies at the same concentration. When the antibody concentration was 600 μg/mL, the inhibition of invasion rates of the anti-rEtAMA1 and anti-rEtESP, or anti-rEtAMA1 and anti-rEtRON2-treatment groups were 21% and 21.3%, respectively, which were higher than those of non-immunized IgGs (*p* < 0.05) (Fig. 3b).

### *In vitro* sporozoite invasion of DF-1 cells transiently transfected with EtAMA1

**Figure 4:**
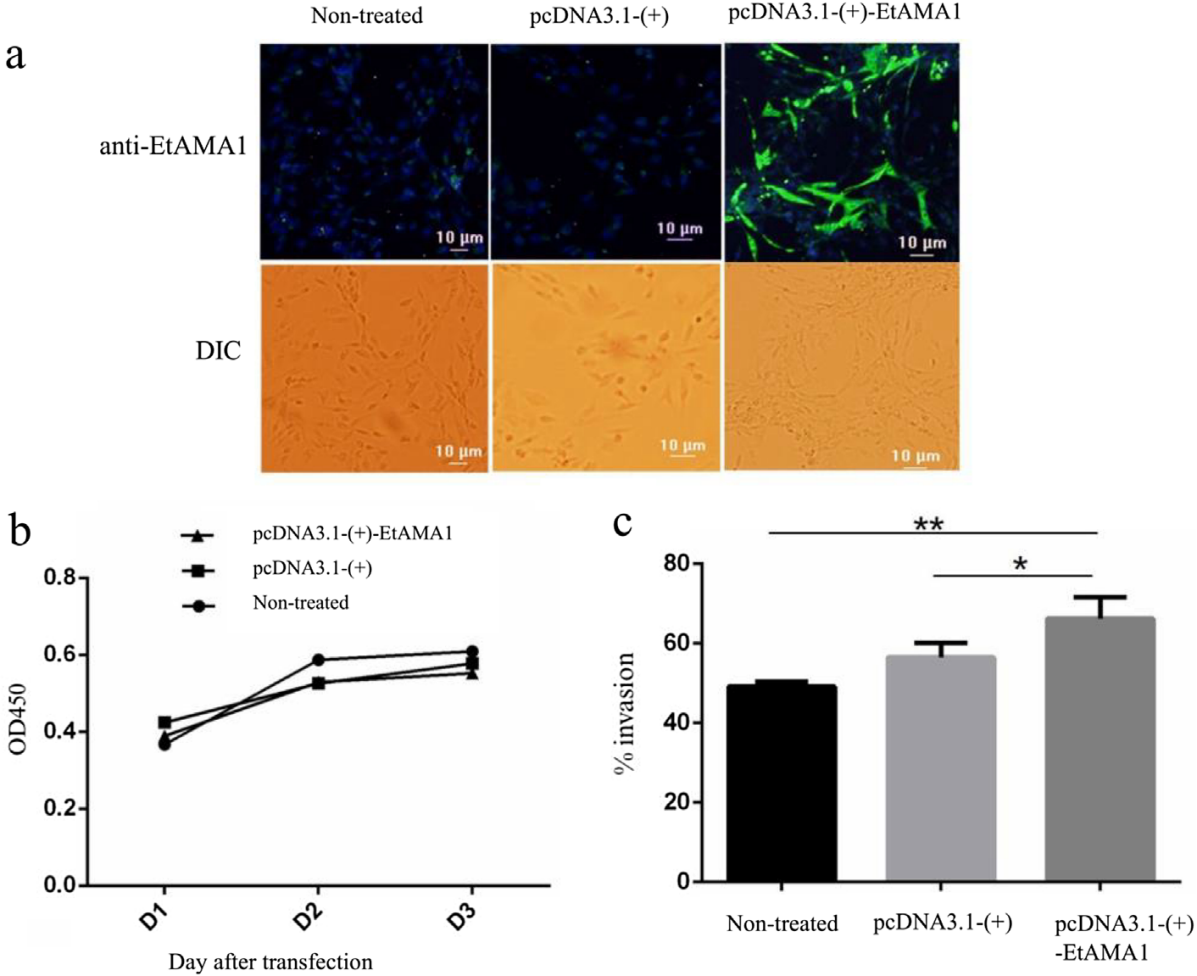
*In vitro* sporozoite invasion of DF-1 cells transiently transfected with EtAMA1. (a) Verification of pcDNA3.1-(+)-EtAMA1 expression in DF-1 cells by IFA. (b) The proliferation of DF-1 cells transfected with pcDNA3.1-(+)-EtAMA1 or pcDNA3.1-(+). (c) Sporozoite invasion rate in DF-1 cells transfected with pcDNA3.1-(+)-EtAMA1 or pcDNA3.1-(+). *p < 0.05 and **p < 0.01, as determined by the Student’s *t*-test versus the untreated group.

To evaluate the effect of EtAMA1 up-regulation on sporozoite invasion, the recombinant plasmid, pcDNA3.1-(+)-*Et*AMA1 was transiently transfected into the DF-1 cells and its expression was confirmed by IFA. Intense green ﬂuorescence was detected in DF-1 cells transfected with pcDNA3.1(+)-EtAMA1 and none was detected in cells transfected with pcDNA3.1(+) (Fig. 4a); thus, demonstrating that pcDNA3.1(+)-EtAMA1 was expressed in DF-1 cells. The proliferation of cells expressing the recombinant pcDNA3.1-(+)-*Et*AMA1 plasmid was similar to that of the control groups (Fig. 4b). Sporozoite invasion assays showed that the sporozoite invasion rate of cells expressing the recombinant pcDNA3.1-(+)-*Et*AMA1 plasmid was significantly higher than that of cells expressing the pcDNA3.1-(+) plasmid (*p* < 0.05), and extremely significantly higher than that of the normal (un-treated) cells (*p* < 0.01) (Fig. 4c).

### Profiling of differentially expressed proteins in DF-1 cells transiently transfected with EtAMA1

After processing the MS/MS spectra using Mascot software, 16,385 unique peptides were mapped to 3953 proteins from DF-1 cells. A total of 163 proteins were found to be signiﬁcantly differentially expressed in DF-1 cells in response to transient transfection with EtAMA1 (Supplementary Table S1). This included 91 upregulated proteins and 72 downregulated proteins. The top ﬁve upregulated proteins were the glucagon like peptide 2 receptor, the protein transport protein Sec61 subunit beta, keratin (type II cytoskeletal cochleal), Wnt-11, and heat shock protein 25. The top ﬁve downregulated proteins were the coiled-coil domain-containing protein 186, the pleckstrin homology domain-containing family O member 1, the 40S ribosomal protein S24, the signal transducer and activator of transcription, and an uncharacterized protein. Collectively, these results indicated that EtAMA1-transfection induced a distinct proteomic proﬁle in DF-1 cells, which caused the host cells to sharply alter their related proteins in response.

**Figure 5:**
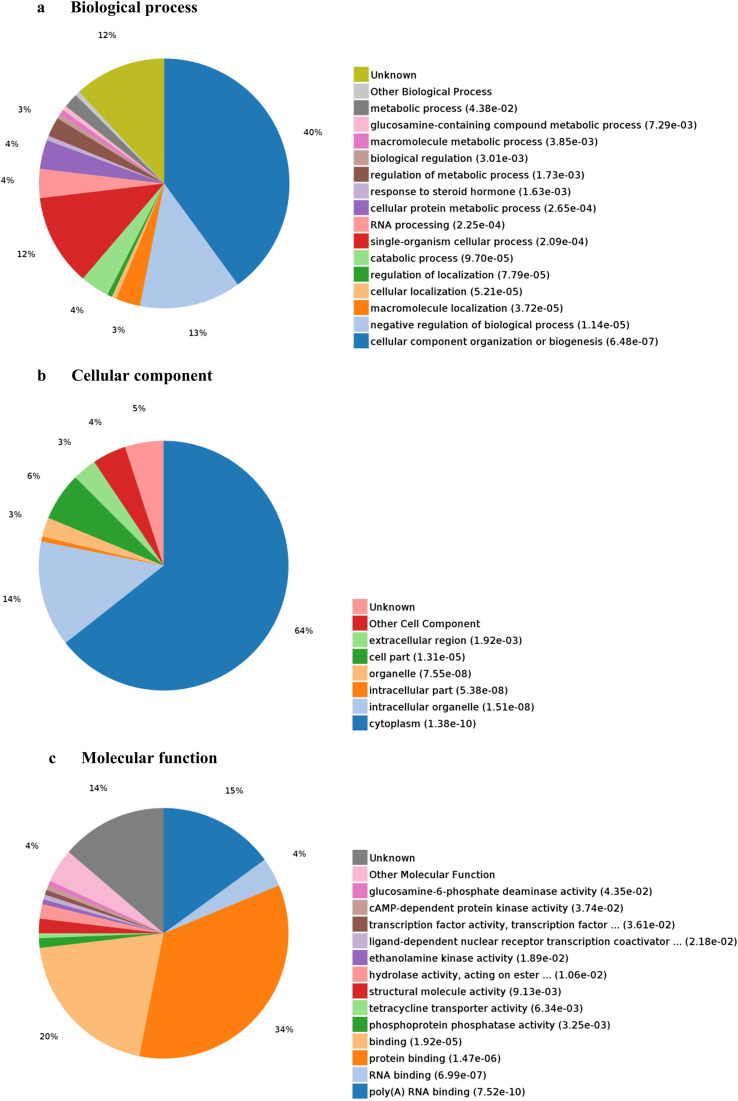
Gene ontology analysis of 163 proteins differentially expressed in DF-1 cells transiently transfected with EtAMA1. Proteins were annotated based on biological process, cellular component, and molecular function.

**Figure 6:**
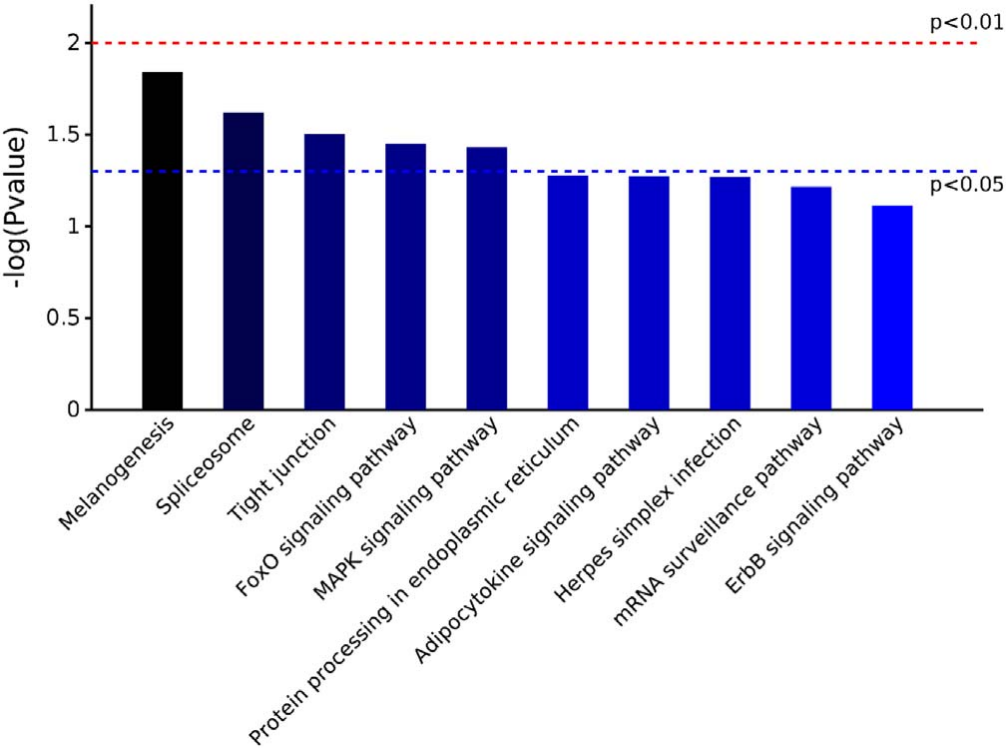
KEGG pathway classiﬁcation of differentially expressed proteins in DF-1 cells transiently transfected with EtAMA1.

GO analyses were used to identify the function of DEPs. The 163 DEPs were categorized into several main biological processes, including cellular component organization or biogenesis (40%), the negative regulation of biological processes (13%), and single organism cellular processes (12%) (Fig. 5a). Additionally, some of the proteins were predicted to be cytoplasmic (64%) or intracellular (14%) (Fig. 5b). Moreover, proteins were assigned to protein binding (34%), binding (20%), and poly(A)-RNA binding (15%) categories (Fig. 5c). KEGG pathway analyses of the DEPs showed that they were involved in melanogenesis, the spliceosome, tight junctions, and the FoxO and MAPK signaling pathways (Fig. 6).

## Discussion

The full-length cDNA of EtAMA1 was 2349 bp, with a 1608 bp open reading frame encoding a protein of 535 amino acids. Analysis of the amino acid sequences revealed that EtAMA1 is a type I integral membrane protein with a transmembrane domain, a signal peptide, an ectodomain, a cytoplasmic C terminal domain, and an AMA1 superfamily domain [18]. EtAMA1 also contained the 16 invariant Cys residues that are encoded in the ectoplasmic region of all characterized apicomplexan AMA1 proteins [11, 30] and contribute to disulfide binding. The pattern of these 16 Cys residues prompted the suggestion that the mature ectodomain folds as an N-terminal pro-sequence and three domains (DI, DII, and DIII) [30]. In the present study, secretion assays showed that EtAMA1 was secreted when sporozoites were incubated at 41 °C in the presence of FCS. Additionally, secretion was inhibited by staurosporine, a protein kinase inhibitor known to speciﬁcally inhibit microneme secretion in *Toxoplasma gondii* and the secretion of EtMIC3 [9, 19]. These results indicate that EtAMA1 is a microneme protein secreted by sporozoites, and its secretion is regulated by FCS and temperature. Our findings are also consistent with a prior study [29].

Comparison of RNAseq datasets from unsporulated oocysts, sporulated oocysts, sporozoites, 2nd-generation merozoites, and the gametocyte lifecycle stages of *E. tenella* had shown that EtAMA1 transcription was stage-speciﬁc [31, 39]. The polyclonal mouse serum raised against rEtAMA1 detected native EtAMA1 in sporulated oocysts and sporozoites, but not in merozoites, thus indicating that EtAMA1 is a sporozoite-specific protein [29]. Immunoﬂuorescent antibody staining of sporozoites with mouse anti-rEtAMA1 showed an apical localization within sporozoites [18, 29]. Blocking EtAMA1 by antibodies, recombinant proteins, or small peptides can significantly inhibit *E. tenella* sporozoite invasion in host cells [18, 23, 29]. To examine whether *E. tenella* sporozoite invasion was also regulated by the overexpression of EtAMA1, invasion experiments were performed with EtAMA1-transfected DF-1 cells. The results showed that the sporozoite invasion of EtAMA1-transfected DF-1 cells was significantly higher than that of the control group, thus indicating that the overexpression of EtAMA1 could promote sporozoite invasion.

Previous studies showed that in *T. gondii* and *Plasmodium*, AMA1 interacts directly with rhoptry neck protein 2 (RON2), which is secreted from the parasite rhoptries and specifically localizes to the MJ. The RON2-AMA1 interaction is a critical step in MJ-dependent invasion of host cells by apicomplexan parasites [8, 37]. To understand the precise functions of EtAMA1 during host-cell invasion, Han et al. screened EtAMA1-interacting proteins in *E. tenella* sporozoites, and a total of 14 putative interaction proteins were obtained [16]. Some putative EtAMA1-interacting proteins were selected to confirm their interaction with EtAMA1 using BiFC and GST pull-down assays, and it was found that *E. tenella* rhoptry neck protein 2 (EtRON2), microneme protein 2 (EtMIC2), and *Eimeria*-specific protein (EtESP) interacted with EtAMA1 [41, 44]. In the present study, IFAs showed that the distribution of EtAMA1, EtESP, and EtRON2 in sporozoites was similar (at the apical end of sporozoites), further verifying their interaction. When anti-rEtAMA1, anti-rEtESP or anti-rEtRON2 IgGs were incubated with sporozoites, their invasion decreased significantly, indicating that these three proteins were involved in sporozoite invasion. However, combinations of anti-rEtAMA1 with anti-rEtESP, anti-rEtAMA1 and anti-rEtRON2 at a ratio of 1:1 exhibited less inhibition than that of the individual antibodies at the same concentration. These data suggested that the combination of different functional antibodies could not provide a more potent reduction in invasion than single antibodies. Our findings are inconsistent with the previous study [26].

EtAMA1 plays an important role in sporozoite invasion, but its molecular mechanism remains unknown. To investigate the effect of EtAMA1 on host cells, the proteomic changes of transfected DF-1 cells were investigated using iTRAQ. iTRAQ has become a powerful quantitative proteomic method with advantages over traditional proteomic techniques. Such advantages include higher throughput, increased sensitivity, and greater accuracy. iTRAQ has been used successfully to explore host-pathogen interactions for viruses [15, 40], bacteria [48], and parasites [46]. The results of proteomic analyses showed that a total of 163 proteins were signiﬁcantly differentially expressed in DF-1 cells after being transiently transfected with EtAMA1. GO analyses showed that 73% of DEPs were involved in binding, including protein binding, RNA binding, and poly (A)-RNA binding. Thus, the data indicated that host binding was strongly affected by transient transfection with EtAMA1.

Previous reports showed that transiently-expressed heterologous proteins could significantly change cell proteomes, and in particular, proteins involved in different pathways [12, 36]. In the present study, KEGG analyses showed that multiple DEPs were involved in MAPK, FoxO, Wnt, Adipocytokine, ErbB GnRH, and AGE-RAGE signaling pathways. These networks included eight DEPs, including four up- and four downregulated proteins. Tropomyosin alpha-1 chain (TPM1), heat shock protein 70 (HSP70), dual specificity protein phosphatase 4 (DUSP4), and Wnt-11 (WNT11) were upregulated in DF-1 cells transfected with EtAMA1. Such upregulations may impact actin cytoskeleton organization, cellular responses, and dephosphorylation. Signal transducer and activator of transcription (STAT2), calcium/calmodulin dependent protein kinase II alpha (CAMK2A), muscle RAS oncogene homolog (MRAS), and TANK binding kinase 1(TBK1) were downregulated. Such downregulations may impact cell signal transducer activity, regulation of metabolic processes, cytoskeletal reorganization, and cell survival. Hence, we predicted that the transfection of EtAMA1 could activate specific signaling pathways in host cells, which is critical for parasite invasion and development.

The cytoskeleton is a cellular scaffolding contained within the cytoplasm. It maintains cell shape, provides mechanical strength, directs locomotion, regulates chromosome separation during mitosis and meiosis, and regulates the intracellular transport of organelles in cells [14]. In the context of *Eimeria* infections, changes in the cytoskeleton of the host cells affect cell adhesion and migration, and may play an important role in the invasion and development of *Eimeria* spp. In the present study, several cytoskeleton-related proteins, including TPM1, alpha-actinin-1 (ACTN1), myosin regulatory light chain interacting protein, septin-2, and keratin were significantly upregulated by the transfection of EtAMA1. Such upregulations may impact cytoskeletal organization, and cell migration and movement tendency. These observations clearly indicated that changes in the cytoskeleton of cells affect cell adhesion and migration, which may play an important role in the invasion and development of *Eimeria* spp.

In conclusion, the present study revealed that EtAMA1 was secreted by micronemes. Both rEtAMA1-specific antibodies and its interacting proteins, EtESP, EtRON2, inhibited parasite invasion, and the overexpression of EtAMA1 in cells promoted sporozoite invasion, thus suggesting that EtAMA1 was involved in host-cell sporozoite invasion. The proteomic changes in EtAMA1-trancfected DF-1 cells were analyzed using an iTRAQ-based proteomic method. Our analyses of the DEPs were comprehensive, but further studies are necessary to understand the functions of the identified proteins regulated by EtAMA1 in DF-1 cells. Such studies will be important to understand the precise functions and molecular mechanisms of EtAMA1 during *Eimeria* host-cell invasion.

## Supplementary Material

Supplementary material is available at https://www.parasite-journal.org/10.1051/parasite/2020068/olm *Table S1*. Detailed list of proteins of DF-1 cells transfected transiently with EtAMA1.
Detailed list of 163 proteins differentially expressed in DF-1 cells transfected transiently with EtAMA1.

## Funding

This work was supported by grants from the National Science Foundation of China (Nos. 31672551 and 31572266), the National Key R&D program of China (No. 2017YFD0500400), and the National Parasitic Resources Center (No. NPRC-2019-194-30).

## Conflicts of interest

The authors declare that they have no competing interests.
